# Soil organic phosphorus transformations during 2000 years of paddy-rice and non-paddy management in the Yangtze River Delta, China

**DOI:** 10.1038/s41598-017-10071-0

**Published:** 2017-09-07

**Authors:** Xiaoqian Jiang, Wulf Amelung, Barbara J. Cade-Menun, Roland Bol, Sabine Willbold, Zhihong Cao, Erwin Klumpp

**Affiliations:** 1 Institute of Bio- and Geosciences, Agrosphere Institute (IBG-3), Forschungszentrum Jülich GmbH, Jülich, 52428 Germany; 2 Institute of Crop Science and Resource Conservation, Soil Science and Soil Ecology, Nussallee 13, University of Bonn, Bonn, 53115 Germany; 3 Swift Current Research and Development Centre Agriculture and Agri-Food Canada Box 1030, 1 Airport Rd., Swift Current, SK S9H 3X2 Canada; 4 Central Institute for Engineering, Electronics and Analytics, Analytics (ZEA-3), Forschungszentrum Jülich GmbH, Jülich, 52428 Germany; 5 Institute of Soil Science, Chinese Academy of Sciences, Nanjing, 210008 China; 6 Present Address: Department of Natural Resources and Environmental Sciences, University of Illinois at Urbana-Champaign, Urbana, IL 61801 USA

## Abstract

The contents and properties of soil organic phosphorus (P_o_) largely drive ecosystem productivity with increasing development of natural soil. We hypothesized that soil P_o_ would initially increase with paddy management and then would persist under steady-state conditions. We analyzed soils from a 2000-year chronosequence of a rice-wheat rotation and an adjacent non-paddy 700-year chronosequence in Bay of Hangzhou (China) for their P_o_ composition using solution ^31^P-NMR after NaOH-EDTA extraction. Land reclamation promoted P_o_ accumulation in both paddy and non-paddy topsoils (depths ≤ 18 cm) until steady-state equilibria were reached within 200 years of land use. Greater P_o_ concentrations were found, however, in the non-paddy subsoils than in those under paddy management. Apparently, the formation of a dense paddy plough pan hindered long-term P_o_ accumulation in the paddy subsoil. The surface soils showed higher proportions of orthophosphate diesters under paddy than under non-paddy management, likely reflecting suppressed decomposition of crop residues despite elevated microbial P compounds stocks under anaerobic paddy-rice management. Intriguingly, the composition of P_o_ was remarkably stable after 194-years of paddy management and 144-years of non-paddy management, suggesting novel steady-state equilibria of P dynamics had been reached in these man-made ecosystems after less than two centuries.

## Introduction

Phosphorus is an essential macronutrient for all life on earth. In soil, there is increasing evidence that the P pool changes its composition, abundance, and bioavailability during soil development in natural and agricultural ecosystems^1–5^. In undisturbed terrestrial ecosystems, organic P (P_o_) accumulates under nitrogen (N) limitation in the early stage of ecosystem development, while at advanced stages of ecosystem development P_o_ concentrations decline under conditions of P limitation^2, 6, 7^. Much less is known, however, about human impacts on temporal soil P dynamics. These impacts might be particularly large in anthropogenically-modified soils such as paddy soils. More than 50% of the world’s population relies on rice as a dietary staple and about 163 million ha of land is used as flooded lowland rice fields^8^. These paddy systems are known to accumulate organic matter and microbial N residues^9–14^, and thus may potentially accumulate P_o_^3^.

Paddy soils environments likely behave differently from natural wetlands, because of the addition of chemical and organic (manure, residues) N and P fertilizers, removal of nutrients during harvest, intensive puddling, and alternating flooding and draining regimes^3, 15^. These processes result in accelerated soil weathering^15^ so that after only 700- to 1000- years, the soils are decalcified and have significant iron (Fe) oxide transformations, which can alter the sorption of specific P_o_ forms^14, 16^.

Soil P_o_ is comprised of a variety of compounds that differ in their stability and biological availability in environment^17^. The variation of P composition over pedogenic time scales has ecological significance^5^, as it may alter species composition of the seedling community, change the competitive ability of adult plant species^18^, and modulate soil microbial P cycling^19, 20^. To reveal changes in P_o_ composition, solution^31^P-nuclear magnetic resonance (^31^P-NMR) spectroscopy after NaOH-Na_2_EDTA extraction has been the method of choice^21–23^. This allows the identification of specific P_o_ species, such as deoxyribonucleic acid (DNA), α- and β-glycerophosphate, mononucleotides, phosphonates, and even inorganic long-chain polyphosphate and pyrophosphate structures, including compounds originating from living microbial cells and fungal tissue^5, 24–26^. However, little information is available on the degree and rates at which different P_o_ species reflect impacts of prolonged paddy or non-paddy management on the overall P_o_ status of the soils.

The objective of this study was to evaluate how prolonged paddy management influenced the degree and rates at which various P_o_ compounds accumulated in soil. For this purpose, we extracted P_o_ with NaOH-Na_2_EDTA and characterized compounds by solution ^31^P-NMR spectroscopy. Samples originated from a unique 2000-year-old chronosequence of paddy soil management; adjacent sites under non-paddy management for 700 years served as a reference. All soils had developed from tidal wetland sediments in the Yangtze River delta, China. We hypothesized that paddy management accelerated the accumulation of P_o_ compounds in soil, which may then persist for several hundred years under steady-state conditions.

## Results

During paddy soil management, the puddling of surface soil accompanies the formation of a dense plough (Ardp) horizon, which potentially restricts root growth into the deeper subsoil^27^. With increasing time of paddy soil development, as these Ardp horizons mature, topsoil surfaces may become increasingly decoupled from subsoil biogeochemistry^13, 14^. In order to understand the role of paddy management in the biological cycling of P, particularly when compared to native ecosystems, we thus have to understand both the transformation of P with increasing soil depth as well as with time of management.

### Changes in phosphorus speciation with soil depth

Total P concentrations decreased with increasing depth from ~800 mg P kg^−1^ in the 100-year-old paddy topsoils and from ~1100 mg P kg^−1^ in the respective non- paddy topsoils down to 40–50 cm soil depth (600–690 mg P kg^−1^) and then remained relatively constant in the subsoil (Table 1). The concentrations of NaOH-Na_2_EDTA extractable total P (E_P_) and inorganic P (E_P_-P_i_) in these soils showed a similar trend (Table 1). The contributions of extracted P_o_ (E_p_-P_o_) to total P in the topsoil of the paddy soil were almost double those under non-paddy management, whereas those in paddy subsoil were reduced compared with subsoil under non-paddy management (Table 1). The contribution of E_p_-P_o_ declined with depth while that of E_p_-P_i_ increased (Table 1). As a result, all P_o_ species in the paddy subsoil (from 30 to 100 cm) showed very low ^31^P-NMR signal intensities, and the spectra were dominated by orthophosphate P (Table 2). The non-paddy site showed a greater proportion of total P as orthophosphate monoesters below 30 cm soil depth than the paddy site, but the lowest subsoil was again dominated by P_i_ (Table 2).

**Table 1:** Bulk density and concentrations of total P (P-total), NaOH-Na_2_EDTA extracted total P (E_p_), organic carbon (OC) and total nitrogen (N-total) at different soil depths after 100 years of paddy and non-paddy management.

Site	Depth	Horizon	Bulk density	OC	N-total	P-total	E_P_	E_p_-P_i_	E_p_-P_o_
	cm		(g cm^−3^)	mg g^−1^	mg g^−1^	mg kg^−1^	mg kg^−1^	% of E_P_	% of E_P_
PR100	0–9	Alp1	1.00	17.6 ± 1.3	2.01 ± 0.24	850	19.1	42.4	57.2
	9–15	Alp2	1.23	15.3 ± 1.1	1.82 ± 0.21	810	18.0	49.9	50.5
	15–21	Ardp	1.53	6.6 ± 0.9	0.84 ± 0.20	780	16.5	43.8	56.7
	21–30	Bwg1	1.55	5.8 ± 0.7	0.55 ± 0.11	640	7.0	61.5	38.4
	30–50	Bwg2	1.48	4.7 ± 0.2	0.43 ± 0.02	600	5.0	73.5	26.7
	50–75	Bwlg1	1.28	4.7 ± 0.1	0.40 ± 0.01	650	5.5	79.1	20.8
	75–100	Bwlg2	1.3	5.2 ± 0.1	0.42 ± 0.01	660	5.4	93.4	6.6
NPR100	0–14	Ap1	1.35	10.8 ± 1.0	1.12 ± 0.22	1100	17.2	77.7	22.5
	14–25	Ap2	1.38	8.1 ± 0.3	0.79 ± 0.06	830	15.7	82.2	17.9
	25–30	Bw	1.47	7.0 ± 0.2	0.65 ± 0.01	850	12.5	66.3	33.9
	30–38	BCwg1	1.47	6.2 ± 0.3	0.55 ± 0.02	690	7.2	60.5	39.3
	38–70	BCwg2	1.51	5.5 ± 0.3	0.47 ± 0.05	720	6.8	58.5	41.4
	70–100	BCwlg	1.49	5.3 ± 0.3	0.48 ± 0.04	670	7.8	79.2	21.4

PR100: 100-year-old paddy soil; NPR100: 100-year-old non-paddy soil.

**Table 2:** Proportions of phosphorus compounds of the total phosphorus extracted by NaOH-Na_2_EDTA in 100-year-old paddy and non-paddy soil with different depths determined by solution ^31^P-NMR spectroscopy.

Site	Depth (cm)	Inorganic P			Organic P							
		(%)										
		Orth	Pyro	Poly	Orthophosphate Monoesters				Orthophosphate Diesters			Phon
					Monoesters*	*myo*-IHP	*Scyllo-*IHP	Other Monoesters	Diesters*	DNA	Glyc + nucl	
PR100	0–9	38.8	2.2	1.4	32.9	7.6	1.9	23.4	23.6	6.5	17.1	0.7
	9–15	45.6	1.7	2.6	26.6	4.7	1.8	20.1	18.8	5.7	12.3	5.1
	15–21	43.8	0.0	0.0	37.8	5.9	2.0	29.9	16.7	0.8	13.1	2.2
	21–30	54.1	0.0	7.4	22.1	1.8	1.2	19.1	10.4	0.7	8.3	5.9
	30–50	70.9	0.0	2.6	6.6	1.2	0.0	5.4	8.0	0.7	5.4	12.1
	50–75	72.9	0.0	6.2	14.6	0.0	0.0	14.6	4.1	0.2	0.0	2.1
	75–100	90.3	2.3	0.8	4.3	0.0	0.0	4.3	0.0	0.0	0.0	2.3
NPR100	0–14	76.3	0.4	1.0	13.5	2.2	0.9	10.4	6.8	0.4	5.8	2.1
	14–25	80.8	0.9	0.5	12.3	2.0	1.4	8.9	3.4	0.0	3.4	2.2
	25–30	63.4	0.5	2.4	21.6	2.7	2.0	16.9	10.1	0.5	7.9	2.2
	30–38	49.8	0.0	10.7	27.8	3.6	1.2	23.0	9.0	0.5	7.3	2.5
	38–70	57.1	1.4	0.0	34.3	0.0	0.0	34.3	2.1	0.8	0.0	5.0
	70–100	77.0	0.0	2.2	14.2	2.0	0.0	12.2	5.0	2.4	1.4	2.2

^*^Calculation by including diester degradation products (α glycerophosphate, β glycerophosphate, and mononucleotides) with orthophosphate diesters (Diesters) rather than orthophosphate monoesters (Monoesters). PR100: 100 year old paddy soil; NPR100: 100 year olds non-paddy soil. Phosphorus compounds include Orthophosphate (Orth), Pyrophosphate (Pyro), Polyphosphate (Poly), *myo-*inositol hexakisphosphate (*myo*-IHP), *scyllo-*inositol hexakisphosphate (*scyllo*-IHP), monoesters other than specifically identified (other monoesters), deoxyribonucleic acid (DNA), α/β glycerophosphate (Glyc), mononucleotides (nucl) and Phosphonates (Phon).

Above 30 cm depth, orthophosphate diesters and *myo*-IHP were much higher proportions of E_P_ in the paddy soils than in the non-paddy soils. There was a substantial increase in phosphonates at the 30–50 cm depth in the paddy soils. Overall, the effects of paddy management on P_o_ dynamics were thus mainly detectable for the surface soils. Hence, we hereafter concentrated on topsoil samples (~0–15 cm) in our study of P transformations along the chronosequence.

### Changes in phosphorus speciation with prolonged management

The tidal wetland (TW) sediment, i.e. the parent material of soil formation for paddy and non-paddy soils, had a total P concentration of 760 mg P kg^−1^ in surface soil (Table 3). After land embankment, soil formation started with the salt marsh (SM) having a comparable P concentration (730 mg P kg^−1^) in the surface horizon. With prolonged arable management, total P concentrations increased, reaching on average significantly greater concentrations in the non-paddy topsoils (average of 1128 ± 198 mg P kg^−1^) than in those under paddy management (average of 890 ± 149 mg P kg^−1^; Table 3; *p* < 0.001).

**Table 3:** Bulk density and concentrations of total P (P-total) and NaOH-Na_2_EDTA extracted P (E_P_), inorganic (E_p_-P_i_) and organic P (E_p_-P_o_) concentration (kg P ha^−1^ and mg P kg^−1^) as well as their respective contributions to P-total or to E-P (%) in soils during 2000 years paddy and 700 years non-paddy management (n ± SD).

Site	Depth cm	Horizon	Bulk density	P-total		E_P_		E_P_ -P_i_		E_P_ -P_o_		E_P_	E_P_ -P_i_	E_P_ -P_o_
			g cm^−3^	kg ha^−1^	mg kg^−1^	kg ha^−1^	mg kg^−1^	kg ha^−1^	mg kg^−1^	kg ha^−1^	mg kg^−1^	% of P-total	% of E_p_	
TW(n = 1)	2–30		1.00	2128	760	90	32	79.9	28.5	9.8	3.5	4.2	89.2	10.9
SM(n = 1)	0–13	Ah	1.32	1253	730	65	38	48.9	28.5	16.4	9.6	5.2	75.0	25.2
PR50(n = 2)	0–14	Alp/Arp	1.11 ± 0.05	1244 ± 77	800 ± 14	150 ± 22	96 ± 10	97.0 ± 10.2	62.3 ± 3.8	52.3 ± 12.0	33.5 ± 6.2	12.0	65.1	34.8
PR100(n = 3)	0–15	Alp	1.15 ± 0.08	1560 ± 22	900 ± 31	269 ± 56	157 ± 39	143.8 ± 56.5	84.2 ± 36.0	125.2 ± 7.0	73.1 ± 8.3	17.4	52.1	47.8
PR300(n = 3)	0–18	Alp	1.17 ± 0.08	2053 ± 224	1000 ± 46	375 ± 59	178 ± 18	218.0 ± 47.3	102.9 ± 14.9	156.4 ± 32.2	74.3 ± 15.4	17.8	58.0	41.8
PR700(n = 2)	0–16	Alp	1.12 ± 0.02	1511 ± 62	800 ± 21	389 ± 28	218 ± 12	284.1 ± 21.3	158.8 ± 9.4	105.0 ± 6.4	58.7 ± 2.6	27.3	73.0	27.0
PR1000(n = 3)	0–16	Alp/Al(d)p	1.24 ± 0.03	1480 ± 139	700 ± 55	401 ± 12	201 ± 2	259.1 ± 30.2	130.1 ± 12.7	141.0 ± 17.5	71.0 ± 10.2	28.7	64.6	35.3
PR2000(n = 1)	0–15	Alp	1.10	2145	1300	673	408	564.8	342.3	110.4	66.9	31.4	83.9	16.4
NPR50(n = 3)	0–17	Ap/ABw	1.34 ± 0.02	2890 ± 651	1300 ± 304	735 ± 240	323 ± 110	617.1 ± 225.1	271.7 ± 102.8	117.8 ± 14.9	51.8 ± 7.4	24.8	83.2	16.8
NPR100(n = 3)	0–14	Ap1	1.30 ± 0.04	1924 ± 259	1100 ± 163	341 ± 202	189 ± 115	255.6 ± 135.9	141.3 ± 77.6	86.3 ± 67.5	48.0 ± 37.9	17.2	77.7	22.5
NPR300(n = 3)	0–11	Ah	1.32 ± 0.02	1724 ± 208	1200 ± 136	440 ± 130	302 ± 87	362.5 ± 119.2	249.1 ± 80.1	77.1 ± 15.4	53.0 ± 9.8	25.2	81.8	18.2
NPR700(n = 3)	0–17	Ap	1.29 ± 0.07	2186 ± 276	1000 ± 91	404 ± 131	187 ± 72	260.4 ± 87.0	120.6 ± 47.5	143.4 ± 48.1	66.5 ± 26.5	18.7	64.4	36.0

TW: tidal wetland; SM: salt marsh; PR50–2000: 50–2000 years paddy soil; NPR50–700: 50–700 years non-paddy soil.

**Figure 1:**
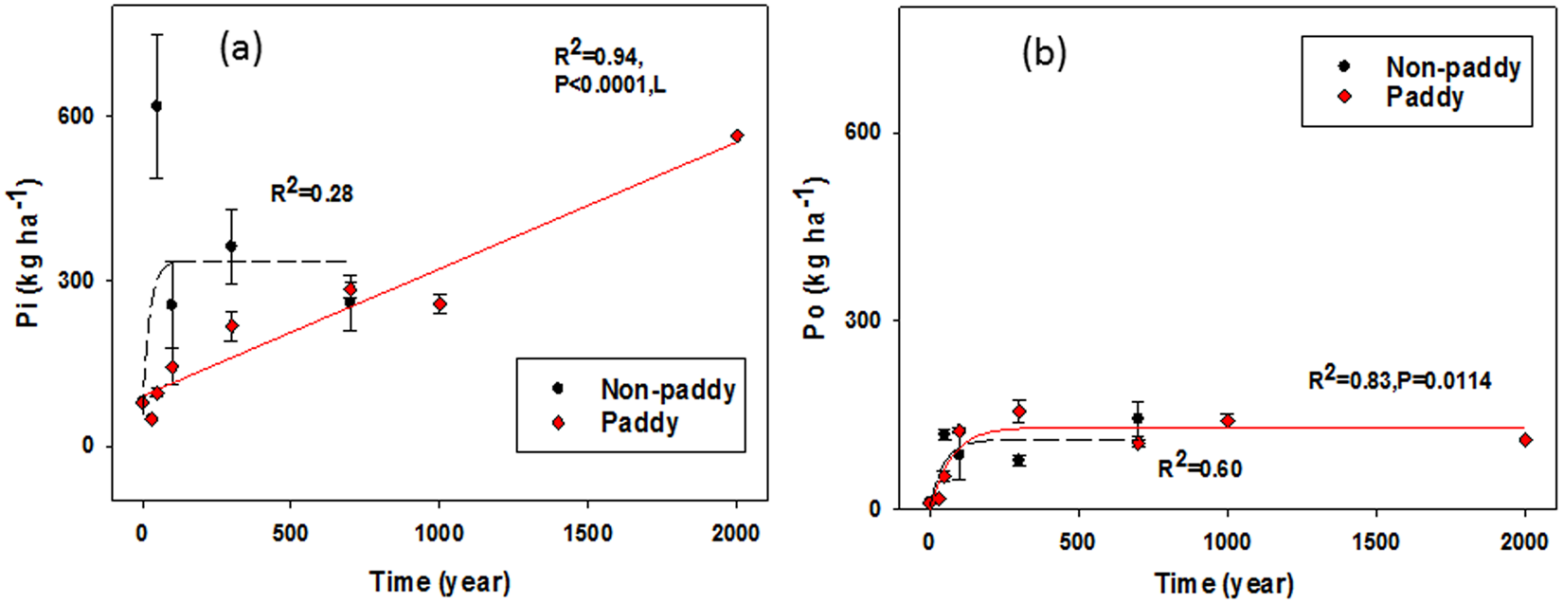
The concentrations (kg ha^−1^) of inorganic (Pi) and organic (P_o_) during 2000 years of paddy and 700 years of non-paddy managements determined in NaOH-Na_2_EDTA extracts by solution ^31^P-NMR spectroscopy (n ± SE). Mono-exponential regression (*P* < 0.05); no significant curvature is indicated by dashed line. Note: at t = 0 year the location was a tidal wetland and after 30 years it was still a salt marsh.

The total P recoveries in NaOH-Na_2_EDTA extracts of the youngest soil were extremely low (4.2–5.2% of total P in TW and SM respectively, Table 3). With prolonged management, the E_P_ concentrations from paddy topsoil increased during pedogenesis to 12.0–31.4% of total P (Table 3). This gain in both absolute concentrations and relative proportions of E_P_ occurred for both P_i_ and P_o_ species, although with different curve progression (Table 3, Fig. 1). Notably, P_o_ concentrations increased only during early stages of soil formation and then remained relatively constant in older soils, both under paddy and non-paddy management (Table 3, Fig. 1b). Kinetic modeling revealed that accumulation of P_o_ in paddy topsoils needed about 50 years more time to reach steady-state (Xe = 178 years) than in non-paddy topsoils, but differences in rate constants (*k*) were not significant when including the uncertainty of *k* into the assessment of Xe (Table 4 and Fig. S1).

**Table 4:** Kinetic parameters of the mono-exponential model (Eqn. 1: *X*_*t*_ = (*X*_*e*_− *X*_0_) × *(1 *− *e*−^*k*^t) + *X*_0_) calculated for different phosphorus (P) pools (see also curve fits in Figs 1 and 2; 2000 years paddy management and 700 years non-paddy management).

Parameters	Unit	*k* ± SE	*X*_0_	*X*_*e*_	Accumulation rate_80_^α^ (1 yr^−1^)	Time to steady-state^β^ (years)	R^2§^
**Paddy**							
P_o_	kg/ha	0.0143 ± 0.0069	—	130.1	0.9490	194	0.83
Poly	%	—	0.0	5.1	**—**	**—**	0.23
P-mono	%	0.0095 ± 0.0088	40.0	55.3	0.1241	113	0.64
Phon	%	0.0187 ± 0.0104	29.8	7.0	—	—	0.77
Myo-IHP	%	0.0289 ± 0.0051	0.1	12.1	0.1738	118	0.97
Scyllo-IHP	%	0.0206 ± 0.0085	0.2	3.5	0.0350	147	0.85
Gly + nucl	%	—	0.0	29.5	—	—	0.85
**Non-paddy**							
Pi	kg/ha	0.0427 ± 0.1012	57.7	336.2	6.3488	85	0.28
P_o_	kg/ha	0.0214 ± 0.0221	3.5	110.6	1.1536	144	0.60
Poly	%	—	0.0	3.3	—	—	0.27
Mono	%	0.0211 ± 0.0287	42.2	60.9	0.3210	91	0.46
Phon	%	0.0307 ± 0.039	29.2	11.0	—	—	0.51
*myo*-IHP	%	0.0343 ± 0.0111	0.0	10.8	0.1833	104	0.94
*scyllo*-IHP	%	0.0346 ± 0.0103	—	4.5	0.0785	104	0.95
DNA	%	—	3.7	1.8	—	—	0.41
Gly + nucl	%	—	0.0	24.7	—	—	0.90

Polyphosphate (Poly), orthophosphate monoesters (Mono), phosphonates (Phon), *myo*-inositol hexakisphosphate (*myo*-IHP), *scyllo*-inositol hexakisphosphate (*scyllo*-IHP), α/β-glycerophosphates and mononucleotides (Glyc + nucl), deoxyribonucleic acid (DNA).

*k*, rate constant; *X*_0_, concentration at time point zero; *Xe*, equilibrium concentration; SE, standard error.

^α^Averaged for the cultivation period until 80% of *Xe* were reached.

^β^Defined as annual increase <0.1% of absolute value of the respective parameter.

**Figure 2:**
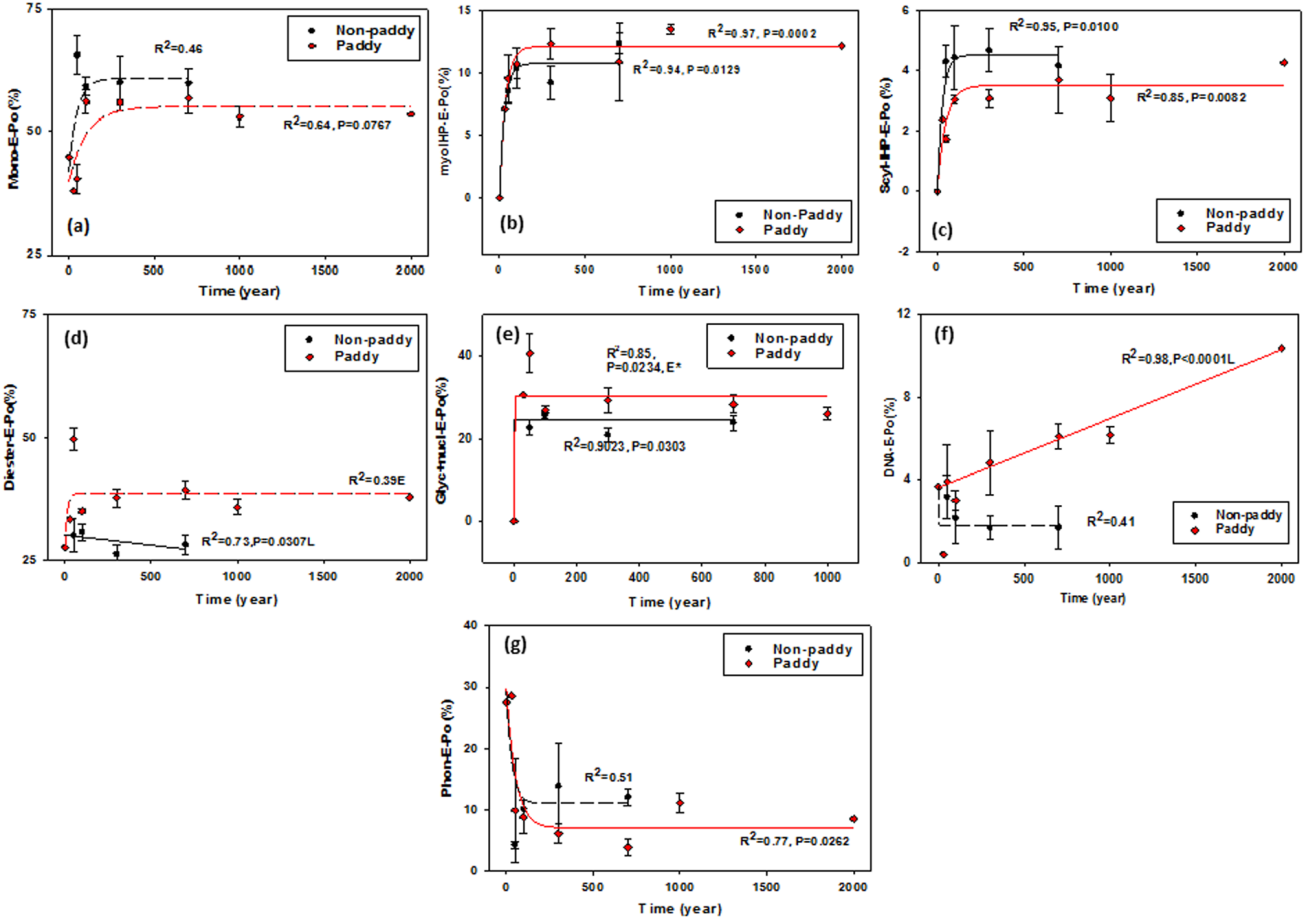
Impact of land-use duration on the proportion of (**a**) orthophosphate monoesters (Mono), (**b**) myo-inositol hexakisphosphate (myoIHP), (**c**) scyllo-inositol hexakisphosphate (scyllo-IHP), (**d**) orthophosphate diesters (Diester), (**e**) α/β-glycerophosphates and mononucleotides (Glyc + nucl), (**f**) deoxyribonucleic acid (DNA) and (**g**) phosphonates (Phon) to total organic P. (n ± SE). Mono-exponential regression (*P* < 0.05); no significant curvature is indicated by dashed line. 0 year, tidal wetland; 30 years, salt marsh; *failed to normality test.

^§^Coefficient of determination of the curve fits (see Figs 1 and 2), mono-exponential regression.

Multiplying the ^31^P-NMR results (in percent) by those for E_P_ and factoring in changes in bulk density gave results in kg P ha^−1^ (Table 5), which showed that concentrations of extracted orthophosphate in paddy soil significantly increased (*p* < 0.001) with increasing duration of paddy rice cultivation. The concentrations of all other P compounds followed a similar trend through early stage of pedogenesis, i.e. being low or undetectable at the onset of soil development in TW and SM, but increasing thereafter in the paddy soils rapidly to a maximum that was obtained after no more than 300 years (Table 5). At the latter stage of the chronosequence, the concentrations of some P compounds like pyrophosphate, polyphosphate, orthophosphate monoesters, *myo*-IHP, other orthophosphate monoesters (calculated as total P in the orthophosphate monoester region after subtracting *myo*-IHP, *scyllo*-IHP, α- and β-glycerophosphate and the mononucleotides) or glycerophosphates + mononucleotides even tended to decline, whereas those of other compounds could be sustained even after prolonged land use (e.g., *scyllo*-IHP, orthophosphate diesters, phosphonates; Table 5). In the non-paddy topsoils, the concentrations of all P compounds increased rapidly after 50 years of management, and then continued to increase slowly (except for orthophosphate and DNA) with subsequent management (up to 700 years; Table 5).

**Table 5:** Concentrations (kg P ha^−1^) of phosphorus compounds during 2000 years of paddy and non-paddy managements determined in NaOH-Na_2_EDTA extracts by solution^31^P-NMR spectroscopy (n ± SD).

Site	Depth cm	Inorganic P			Organic P							
		Orth	Pyro	Poly	Orthophosphate Monoesters				Orthophosphate Diesters			Phon
					Monoesters*	*myo*-IHP	*Scyllo-*IHP	Other Monoesters	Diesters*	DNA	Glyc + nucl	
TW	2–30	78.6	1.3	0.0	0.0	0.0	0.0	4.4	2.7	0.4	0.0	0.0
SM	0–13	44.2	1.2	3.3	0.2	0.2	0.1	4.7	5.5	0.1	0.0	0.3
PR50	0–14	86.5 ± 13.6	2.5 ± 0.7	8.0 ± 2.7	20.9 ± 2.7	5.1 ± 2.5	0.9 ± 0.1	14.9 ± 0.0	25.7 ± 4.2	2.2 ± 1.8	20.9 ± 1.3	5.6 ± 5.1
PR100	0–15	123.8 ± 49.3	5.4 ± 0.3	14.6 ± 7.2	70.5 ± 7.9	13.4 ± 2.6	3.8 ± 0.5	53.2 ± 9.6	43.8 ± 3.0	3.8 ± 1.2	33.8 ± 0.4	11.0 ± 5.8
PR300	0–18	204.0 ± 53.0	6.9 ± 2.9	7.1 ± 6.3	88.0 ± 19.1	19.1 ± 3.5	4.8 ± 0.9	64.1 ± 17.1	58.3 ± 7.0	8.1 ± 6.1	44.8 ± 2.3	10.2 ± 6.2
PR700	0–16	276.0 ± 28.7	2.4 ± 0.7	5.7 ± 8.1	59.9 ± 8.4	11.3 ± 3.9	3.8 ± 1.4	44.7 ± 13.6	41.1 ± 0.4	6.4 ± 1.3	29.6 ± 1.5	4.0 ± 1.6
PR1000	0–16	245.5 ± 28.5	3.7 ± 1.8	9.9 ± 8.9	75.2 ± 13.2	19.0 ± 2.3	4.3 ± 1.6	51.9 ± 11.8	50.1 ± 2.6	8.8 ± 1.9	36.4 ± 1.1	15.7 ± 4.5
PR2000	0–15	559.4	2.7	2.7	59.2	13.5	4.7	41.1	41.7	11.4	26.9	9.4
NPR50	0–17	605.4 ± 225.6	5.0 ± 1.9	6.7 ± 8.3	77.0 ± 8.2	10.3 ± 3.4	5.1 ± 1.7	61.6 ± 8.2	35.7 ± 10.7	3.8 ± 2.6	26.9 ± 6.8	5.1 ± 1.7
NPR100	0–14	250.9 ± 133.1	0.8 ± 0.8	4.0 ± 3.4	52.5 ± 42.5	7.8 ± 4.9	3.0 ± 1.6	41.7 ± 36.7	25.5 ± 18.3	0.9 ± 0.9	22.8 ± 18.7	8.3 ± 7.2
NPR300	0–11	350.0 ± 125.2	5.5 ± 3.5	7.0 ± 5.0	46.5 ± 14.1	6.9 ± 0.4	3.5 ± 0.3	36.1 ± 14.5	20.0 ± 3.2	1.3 ± 0.6	16.1 ± 3.7	10.6 ± 9.0
NPR700	0–17	241.3 ± 76.9	6.5 ± 1.1	12.6 ± 9.7	87.4 ± 37.0	18.2 ± 8.2	6.3 ± 3.7	62.9 ± 25.2	39.3 ± 8.2	2.2 ± 1.8	33.4 ± 7.2	16.8 ± 3.8

*Calculation by including diester degradation products (α glycerophosphate, β glycerophosphate, and mononucleotides) with orthophosphate diesters (Diesters) rather than orthophosphate monoesters (Monoesters). TW: tidal wetland; SM: salt marsh; PR50–2000: 50–2000 years paddy soil; NPR50–700: 50–700 years non-paddy soil. Phosphorus compounds include Orthophosphate (Orth), Pyrophosphate (Pyro), Polyphosphate (Poly), myo inositol hexakisphosphate (myoIHP), scyllo inositol hexakisphosphate (scylloIHP), other monoesters not specifically identified (other monoesters), deoxyribonucleic acid (DNA), α/β glycerophosphate (Glyc), mononucleotides (nucl) and Phosphonates (Phon).

To better understand changes in P dynamics, we normalized the respective concentrations to the total amount of P_i_ or P_o_ extracted, i.e., we evaluated to which degree a given P_i_ or P_o_ compound contributed to overall P_i_ or P_o_ from ^31^P NMR spectra. The results showed that paddy topsoils showed increasing proportions of total Pi for orthophosphate and declining ones for pyrophosphate during soil development, whereas non-paddy topsoils showed the opposite tendency (Fig. S1a and b). The contributions of polyphosphates to extractable P_i_ did not exhibit a clear temporal trend in both land-use systems, though proportions were larger in the paddy soils (Fig. S1c).

In contrast to the P_i_ forms, changes in P_o_ composition were more consistent: the proportions of orthophosphate monoesters to extracted P_o_ increased until a steady-state maximum was reached after 113 years of paddy and 91 years of non-paddy management (Table 4, Fig. 2a). Moreover, orthophosphate monoesters constituted the highest proportion of total P_o_ in both paddy and non-paddy topsoils at steady-state, though with higher proportions in the non-paddy soils (Fig. 2a). The *myo*-IHP and *scyllo*-IHP as typical orthophosphate monoesters showed similar accumulation patterns to total orthophosphate monoester, but they constituted only a small proportion of total P_o_ in both paddy and non-paddy topsoils (Fig. 2b and c, Table 5). The orthophosphate monoesters, *myo*-IHP, and *scyllo*-IHP were found to need a longer time to reach steady-state in paddy topsoils than in non-paddy topsoils (Table 4).

**Figure 3:**
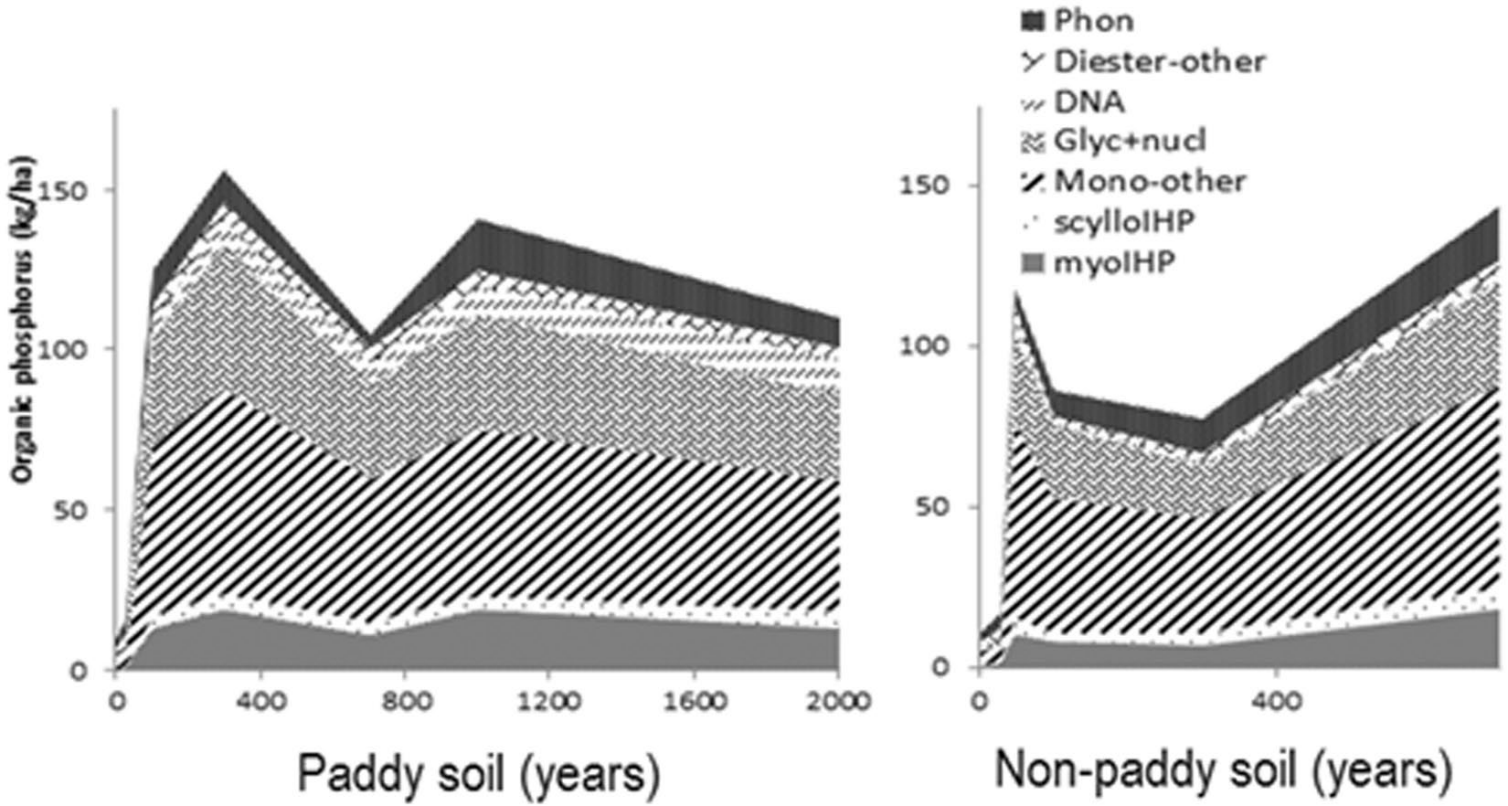
Summary of the changes in organic phosphorus compounds (kg/ha) including *myo*-inositol hexakisphosphate (myoIHP), *scyllo*-inositol hexakisphosphate (scylloIHP) other orthophosphate monoesters (mono-other), deoxyribonucleic acid (DNA), α/β-glycerophosphates and mononucleotides (Glyc + nucl), other orthophosphate diesters (diester-other), and phosphonates (Phon) along the 2000-year paddy soil chronosequence and 700-year non-paddy chronosequence, the Yangtze River Delta, China.

Although orthophosphate diesters may have a different origin than orthophosphate monoesters, the contribution of orthophosphate diesters to total P_o_ also increased rapidly at early stages of pedogenesis until a steady-state plateau was reached in the paddy soils; no such trend was observed for the non-paddy topsoils (Fig. 2d). The sum of the diester degradation products (α/β-glycerophosphates from phospholipids and mononucleotides from RNA) also showed this curve progression to a plateau with higher proportions in the paddy topsoils (Fig. 2e). The DNA content was larger in the paddy topsoils than the non-paddy ones, and tended to increase with the increasing duration of management and related pedogenesis in the paddy soils (Fig. 2f). Only the phosphonates did not confirm a P accrual with time – their proportions declined with time in both paddy and non-paddy soils (Fig. 2g). To determine if the modelling results were affected by the longer time scale for the paddy soils, we redid our analyses using 700 years as the oldest date for both soil types. The trends observed when the 1000 and 2000 year old sites were included for the paddy soils were also observed with the analyzes up to 700 years only (Fig. S2 and Table S2). The overall changes in organic P compounds with time are shown in Fig. 3.

## Discussion

### Changes in soil properties with time

We did not analyze any physical properties for these samples, nor any chemical properties beyond P forms by P-NMR, because this information is available in other studies^13, 15, 28^. We have summarized the information here that is expected to be relevant for understanding P cycling in these soils. The pH values were 8.2 and 7.8 in the youngest soil (TW and SM; Table 6). With prolonged soil development, the pH values declined to 5.1 after 2000 years of paddy-rice management. This increasing acidification was related to the loss of carbonates (Table 6)^14^. In this regard, long-term paddy cultivation with submergence and drainage, as well as ploughing and puddling, resulted in faster decalcification than in non-paddy soils^14^, and thus in accelerated loss of related primary materials (e.g. CaCO_3_), clays, and P-sorbing Fe oxides^3^. These processes likely led to the increase in P in non-paddy soils compared with soils under paddy management (Table 3), despite greater SOM accrual in the latter^13, 14^. Higher yields of paddy rice than non-paddy crops also contributed to lower P storage in paddy soils.

**Table 6:** The pH, total calcium (Ca), inorganic carbon concentrations(IC), dithionite-citrate-bicarbonate extractable iron (Fe_DCB_), and oxalate extractable Fe (Fe_ox_) concentrations of different paddy and non-paddy topsoils.

Site	Depth (cm)	Horizon	pH*	Ca* (mg g^−1^)	IC*^a^ (mg g^−1^)	Fe_DCB_* (mg g^−1^)	Fe_ox_* (mg g^−1^)
TW(n = 1)	2–30		8.2	32.1	5.0	7.38	4.03
SM(n = 1)	0–13	Ah	7.8	31.8	4.2	7.99	3.39
PR50(n = 2)	0–14	Alp/Arp	7.4–7.5	17.1–20.5	1.4–1.6	8.42	4.03
PR100(n = 3)	0–15	Alp	5.0–5.8	7.9–8.2	<0.1	8.70	3.14
PR300(n = 3)	0–18	Alp	5.8	8.6	<0.1	8.86	2.88
PR700(n = 2)	0–16	Alp	6.6–6.7	9.1–10.3	<0.1	7.97	4.33
PR1000(n = 3)	0–16	Alp/Al(d)p	5.2–5.8	7.1–7.3	<0.1	8.29	3.63
PR2000(n = 1)	0–15	Alp	5.1	6.6	<0.1	5.95	3.13
NPR50(n = 3)	0–17	Ap/ABw	7.3	22.1–24.5	1.6–2.2	8.43	2.53
NPR100(n = 3)	0–14	Ap1	7.3	20.4	0.7	8.57	1.85
NPR300(n = 3)	0–11	Ah	7.0	10.7	0.1	7.84	1.14
NPR700(n = 3)	0–12	Ap	5.9–6.6	9.5	<0.1	6.86	2.09

^a^Inorganic carbon; *data from Kölbl *et al*.^15^; TW = tidal wetland; SM = salt marsh, PR = paddy soil, NPR = non-paddy soil.

Concentrations of calcium (Ca), magnesium (Mg), Fe, manganese (Mn), and aluminum (Al) decreased with cultivation time in the paddy topsoils, so that the greatest concentrations of these elements were found mainly in the younger surface soils^15^. The dithionite-citrate-bicarbonate- (DCB-) extractable Fe in paddy and non-paddy chronosequences did not show large differences. However, greater concentrations of oxalate-extractable Fe (i.e. poorly crystalline Fe) in the paddy topsoils than in the non-paddy soils supported previous findings of enhanced redox cycling and accelerated weathering (Table 6). The greater proportion of poorly crystalline Fe oxides was probably also responsible for the large proportion of mineral-associated soil organic matter in paddy soils^29^. The N and microbial residue accumulation were limited to initial stages of paddy soil development and restricted to the surface horizons^13^. Consequently, we assumed similar impacts on related P forms.

### Phosphorus extraction and identification of phosphorus forms

Overall, NaOH-Na_2_EDTA extracted 12–31% of P-total in the paddy and non-paddy soils. We tested extraction methods to try to improve P recovery for these soils (see supplemental information); however, we found that a sequential repeated extraction did not increase the proportion of extracted P_o_ and caused hydrolysis for some orthophosphate diesters (data not shown). The low P extraction yield in these soils may be related to the marine sediment parent materials and inputs of riverine sediments; most labile P was probably removed by sea water, leaving the majority of P occluded within Ca minerals. Low P recoveries (16–29% of total P) have been reported for NaOH-EDTA extracts of Ca-rich sea sediments^30, 31^, and similar low recoveries of P from Ca-rich soils have been reported for NaOH-Na_2_EDTA extracts^32, 33^ and for NaOH-NaF extracts^34^. The total P recoveries in NaOH-Na_2_EDTA extracts of the youngest soils without pronounced pedogenesis were extremely low (4.2–5.2% of total P in TW and SM respectively, Table 3). Turner *et al*.^22^ suggested that low proportions of extracted P indicate that the majority of phosphate is present in primary minerals. The low TP and P_o_ concentrations of the tidal wetland and salt marsh samples are consistent with other reports of P concentrations in the sediments of this region^35^. It is important to note, however, that inputs of riverine sediments to coastal areas in this region have been significantly altered by upstream water diversion over time^36^. As such, current nutrient concentrations in the tidal wetland and salt marsh may not fully reflect concentrations 700 or 2000 years ago, but should reflect conditions for the more recent soils in these chronosequence.

### Organic phosphorus storage in paddy and non-paddy soils

The paddy and non-paddy soils of the chronosequence were formed from marine sediment with an extremely low P_o_ concentration, as indicated by the TW E_P_-P_o_ concentrations (Table 3). This suggests that the majority of P_o_ in the older paddy and non-paddy soils did not originate from marine P. After land embankment, soil formation led to an initial accumulation of P_o_ from the few grasses and shrub vegetation growing in the topsoils^37^. With the start of cultivation, the P_o_ stocks continued to increase exponentially (Fig. 1b), probably due to increased biomass inputs from the crops and manure applications^13^ and from decreasing pH and changes in mineralogy^17^.

The decomposition of organic matter is less efficient under anaerobic conditions during anthropogenic submergence of paddy topsoils. This results in a larger accumulation of organic matter relative to the non-paddy topsoils^14, 27^. The gains in organic matter and the use of organic fertilizers such as manures likely promoted P_o_ accrual in the surface soils^15^. Dense plough pans, developed from anthropogenic ploughing and puddling in the paddy soils^13^, reduced infiltration rates and thus blocked the vertical transport of organic matter into paddy subsoil^38, 39^. As a result, the concentrations of all P_o_ species generally declined more strongly with depth in the paddy soil profile than in the adjacent non-paddy counterpart (calculated by using Tables 1 and 2). We believe that this tendency is consistent with soil profiles of other ages, given that the 100-year-old paddy topsoil had some of the largest P_o_ concentrations among the soils samples along the chronosequence (Table 3). Thus, the increase in paddy P_o_ stocks over time with cultivation seems to be mainly driven by the accumulation of P in the topsoil but not by processes storing P in the subsoils. In addition, advanced Fe reduction in the paddy subsoil^15^ likely hindered the effective binding of P in that part of the soil.

The accumulation of P_o_ in both land-use systems followed exponential growth curves to a maximum, indicating a saturation of P_o_ concentrations and stocks in both systems over time. Such saturation implies both limited sorption capacity and steady-state conditions, i.e., at given amounts of P_o_ stored, P_o_ input and output rates must be similar. The findings are consistent with patterns of organic N accumulation in topsoils of both land-use systems, which reached plateaus within a similar time scales [78–193 years for N stock^13^ and 144–194 years for P_o_ stock].

### Inorganic and organic phosphorus pools

Phosphorus speciation along a chronosequence is mainly regulated by changes in soil mineralogy for P stabilization, P inputs and biological production and utilization of various P compounds^2^. Alkali-extractable P_i_ was classified as phosphate that is mainly associated with Fe and Al in soil^40, 41^. It is a dynamic P pool and may act as an important P source for rice growth^42^. As P_i_ concentrations continuously increased with increasing duration of paddy management (Table 5), our results implied a sustained change in soil nutrient composition for at least 2000 years. The results are different from native ecosystems where fertilization is lacking: results from a coastal dune chronosequence and nearby Franz Josef glacial chronosequence in New Zealand indicated that the concentrations of alkali-extractable phosphate increased only in the early stages of pedogenesis and then declined due to depletion of total P^2, 5^.

The concentrations of pyrophosphate and polyphosphate were so small that they hardly contributed to the overall contents of total P_i_. The proportions of pyrophosphate even decreased with time in paddy soil, suggesting that it is not a stable P form in these environments. Low pyrophosphate proportions were also found in humic acid extracts of paddy soils in Philippines^43^. We conclude that pyrophosphate is easily decomposed, even when microbial activity may be reduced during the wet season under submerged anaerobic conditions in paddy soil^43^. Certainly, these changes in redox conditions promoted greater steady-state percentages of orthophosphate diesters in paddy than in the non-paddy soils (Fig. 2d). Orthophosphate diesters may accumulate due to reduced decomposition or increased microbial synthesis. The exact source assignment of orthophosphate diesters to plants and microbes is frequently ambiguous. However, paddy soils also contained larger contents of microbial N residues than their non-paddy counterparts^13^; Therefore, it seems reasonable to assume co-accumulation of some microbial P forms that were rich in orthophosphate diesters^5^.

Intriguingly, and unlike other P_o_ species, the portions of DNA continued to increase in paddy soils with time (Fig. 2f). The DNA extracted for ^31^P-NMR may be present in the soil in microorganisms or plant residues, or may be stabilized on soil minerals, and stabilization in soil or in soil organic matter may be favored at lower soil pH^2, 17^. Similarly, proportions of DNA increased with time in the 120,000 year Franz Josef post-glacial chronosequence, New Zealand, correlating significantly and positively with soil total organic carbon and N and negatively with soil pH^2^. Thus, with respect to accumulation of DNA with time, both the anthropogenically-modified ecosystems of this study and the native ecosystems in the New Zealand showed similar stabilization patterns of DNA with time.

The proportion of the sum of α/β-glycerophosphate and mononucleotides to total P_o_ increased in the first 50 years and then slightly declined with prolonged cropping (Fig. 2e), which caused a similar tendency for total orthophosphate diesters. However, the tendency is equivocal because the highest proportion at 50 years is only sustained by one data point and may thus also be an outlier in our chronosequence; additionally, no other P species showed a similar tendency. Therefore, we ignored the highest point and considered the α/β-glycerophosphate and mononucleotides to have reached a plateau after steady-state conditions. Soil phospholipids and RNA are associated with living soil microbial biomass^2, 44–46^. In this line, a higher proportion of α/β-glycerophosphate and mononucleotides is consistent with increased microbial residues in the paddy soils compared with non-paddy soils that were observed in previous studies from these sites^13, 15^.

Unlike the forest/shrub soil chronosequences in New Zealand with abundant inositol hexakisphosphate^1, 2, 5^, *myo*-IHP and *scyllo*-IHP constituted only a small proportion of orthophosphate monoesters in our study. In general, inositol phosphates may be stabilized by amorphous Fe oxides^2^, and close relationships between the contents of inositol phosphates and amorphous metal oxides have been found in a range of ecosystems^2, 47–50^. However, the submergence during paddy cultivation induced anaerobic conditions, under which amorphous Fe oxides are easily dissolved. Hence, associated inositol phosphates may be released or even lost with subsequent drainage or surface runoff. Additionally, there could be potentially larger inputs of inositol hexakisphosphate from plant materials (e.g. seeds) into the forest/shrub soil chronosequences in New Zealand than into the studied paddy soils^2^.

### Kinetic response of organic phosphorus to prolonged paddy and non-paddy management

The Walker & Syers model^51^ of P transformation predicts that with increasing time of pedogenesis there will be a loss in total P, a depletion of primary mineral P pools, but an accumulation of P_o_ stocks in early stage of soil development followed by a slow, subsequent decline with time. Our sites are different to those studies by Walker and Syers^51^ in that our soils are fertilized to supplement P losses; besides, our land-use sequence covers a much shorter time scale to the chronosequence of Walker and Syers^51^. Our detailed assessment of P_o_ species using^31^P NMR, including individual P_o_ compounds and functional groups along 2000 years of rice paddy and non-paddy soil development demonstrated that: (i) the chemical nature of P_o_ changes during pedogenesis, and (ii) soil paddy management strongly influences P_o_ composition. All P_o_ species accumulated rapidly in the early stages of pedogenesis. Thereafter, a plateau that is typical for steady-state conditions was reached. Studies in natural ecosystems^2, 6, 7^ noted that P_o_ stocks that accumulated during early soil development stages would decline gradually in later stages of the ecosystem, as a response to nutrient limitation. For example, soil P_o_ accumulated under N limitation but declined under P limitation in natural ecosystems^2^. In our arable study sites, concentrations of available N were low in the young paddy systems^13^, so the initial gain in P_o_ may be explained by similar processes as described by Turner *et al*.^2^; in addition, the mere accrual of organic matter^14^ co-accumulated P_o_. In older systems, however, nutrient limitations have been counterbalanced by fertilization, so P_o_ losses in response to P limitation do not occur because these ‘old’ systems do not become P limited.

## Materials and Methods

### Samples

The studied soils are located in the Bay of Hangzhou near the city of Cixi (30°10′N, 121°14′E), Zhejiang Province. Dike building over last 2000 years for land reclamation resulted in a chronosequence containing paddy and non-paddy soils. More details about the sites are available in other publications^14, 15, 52^. A sample of the marine Yangtze River sediment in a tidal wetland (TW, 2–30 cm) was taken in the Bay of Hangzhou as substrate reference. After 30 years of land embankment behind the youngest dike, built in 1977, an initial surface soil was considered as the starting point (0 years of cropping) for our land use with the growth of salt-tolerant shrubs on an initial salt marsh (SW, 0–13 cm). The soil chronosequence contained replicated sites under paddy cultivation for 50, 100, 300, 700, 1000, and 2000 years (PR50–2000), as well as adjacent replicated sites under non-paddy cultivation for 50, 100, 300, and 700 years (NPR50–700). Additionally, the soils with 100 years paddy and non-paddy managements (i.e. PR100 and NPR100) were sampled by soil horizon to around 100 cm for elucidating changes in P_o_ composition with soil depth; for the other soils we only analyzed the top A horizons (~0–15 cm depth). More details about these soil samples are shown in Table 6 and may also be obtained from Roth *et al*.^13^ and Lehndorff *et al*.^28^. It should be noted that the traditional paddy management practice in this region in China is a crop rotation of rice in the wet season followed by wheat or other upland crops in the dry season^53^. Total soil P concentrations were determined by melting a mixture of soils and lithium borate at 1000 °C for 30 min, followed by inductively coupled plasma optical emission spectrometry (ICP-OES)^54^.

### Organic P analyses

Solution ^31^P-NMR spectroscopy of alkaline extracts is commonly considered to be a reliable method for the quantification of P_o_^40^. Soil samples from each of the replicated plots at each location were extracted by shaking 4 g of air-dried soil with 40 mL of a solution containing 0.25 M NaOH and 0.05 M Na_2_EDTA for 4 h, followed by centrifugation at 10,000 × *g* for 30 min^23, 55^. A 2-mL aliquot of each supernatant was used to determine total extracted P by ICP-mass spectrometer (MS); the remaining solutions were frozen at −80 °C, lyophilized, and ground. Each freeze-dried extract (~100 mg) was re-dissolved in 0.1 mL of deuterium oxide and 0.9 mL of a solution containing 1.0 M NaOH and 0.1 M Na_2_EDTA, and then immediately transferred to a 5-mm NMR tube.

Solution ^31^P-NMR spectra were obtained using a Bruker 600-MHz spectrometer equipped with a prodigy-probe (a broadband CryoProbe that uses N-cooled RF coils and preamplifiers to deliver a sensitivity enhancement over room temperature probes by a factor of 2 to 3 for nuclei from ^15^N to ^31^P). Extracts were measured with a D_2_O-field lock at room temperature, and chemical shifts were referenced to 85% orthophosphoric acid (0 ppm). The NMR parameters generally used were: 32 K data points, 2.6 s repetition delay, 0.7 s acquisition time, 30° pulse width and 10,000 scans. The delay time used here allows sufficient spin-lattice relaxation between scans for P compounds in NaOH-Na_2_EDTA (see supplemental information Fig. S3). Peak areas were calculated by integration on spectra processed with 7 and 2 Hz line-broadening, using NUTS software (2000 edition; Acorn NMR, Livermore, CA) and manual calculation. Individual P compounds were identified by manual inspection and the peak-picking subroutine in the NUTS software, based on their chemical shifts from reports in the literature^56^ and by spiking selected samples with *myo*-inositol hexakisphosphate (*myo*-IHP)^1^. The orthophosphate peak in each spectrum was standardized to 6.0 ppm during processing^57, 58^. Identified compounds and compound classes include orthophosphate (6 ppm), pyrophosphate (~−5 ppm), polyphosphate (−4 to −5, −5 to −50 ppm), orthophosphate monoesters (3 to 6, 6 to 7 ppm), orthophosphate diesters (3 to −4 ppm), and phosphonates (7 to 50 ppm). Because α- and β-glycerophosphates and mononucleotides, which result from degradation of orthophosphate diesters during ^31^P-NMR analysis^59^, were detected in orthophosphate monoester region, we assigned these compounds to orthophosphate diesters rather than to monoesters^58, 60^. The concentrations of individual P species were calculated by multiplying ^31^P-NMR proportions by the total NaOH-Na_2_EDTA extractable P concentration. The percentage of each P compound or compound class was calculated manually by integration across the entire spectrum and also by integrating smaller regions within each spectrum. The high-molecular weight (HMW) P_o_ recently identified by McLaren *et al*.^61^ is included with the unknown peaks in the P-mono-other category (Table S3). In our experience (B. Cade-Menun, unpublished data), peaks for these unknown HMW compounds are better resolved when lyophilized samples are prepared for NMR with NaOH-EDTA than with only water and D_2_O^61^, which is also reflected in the clear separation of all the *myo*-IHP peaks from the orthophosphate peak (Fig. S4).

### Statistical evaluation

The total P concentrations between paddy and non-paddy soils were tested for significant differences (set to *p* < 0.05) by t test, one-way ANOVA was used to test significant differences of P compound concentrations among soil chronosequences, and regression functions of concentrations and distributions of various P_i_ and P_o_ during pedogenesis were calculated. All statistical analyses were conducted using Sigmaplot 12.5 for Windows. To estimate accumulation rates, a mono exponential regression model was used:

1 $${X}_{t}=({X}_{e}-{X}_{0})\times (1-{e}^{-kt})+{X}_{0}$$

where *X*_*t*_ is the parameter of issue at cultivation time t (years), *X*_*e*_ is the parameter stock at absolute equilibrium, *X*_0_ is the initial parameter concentration in the tidal wetland (t = 0), and *k* is a rate constant^13^.

### Data availability

All data generated or analysed during this study are included in this published article (and its Supplementary Information files).

## Electronic supplementary material

supplementary information
