# Supplementary material for: Soil organic phosphorus transformations during 2000 years of paddy-rice and non-paddy management in the Yangtze River Delta, China
Source: Sci Rep. 2017 Sep 7;7:10818. doi: 10.1038/s41598-017-10071-0 (PMC5589726; doi:10.1038/s41598-017-10071-0)
Supplement: Supplementary file 1 — supplementary information [file 41598_2017_10071_MOESM1_ESM.pdf]

Supplement information

Soil organic phosphorus transformations during 2000 years of paddy-rice and non-paddy management in the Yangtze River Delta, China

Xiaoqian Jiang[1*], Wulf Amelung[1, 2], Barbara J. Cade-Menun[3], Roland Bol[1], Sabine Willbold[4], Zhihong Cao[5], Erwin Klumpp[1]

[1] Institute of Bio- and Geosciences, Agrosphere Institute (IBG-3), Forschungszentrum Jülich GmbH, Jülich, 52428, Germany

[2] Institute of Crop Science and Resource Conservation, Soil Science and Soil Ecology, Nussallee 13, University of Bonn, Bonn, 53115, Germany

[3] Swift Current Research and Development Centre Agriculture and Agri-Food Canada Box 1030 1 Airport Rd. Swift Current, SK, S9H 3X2 Canada

[4] Central Institute for Engineering, Electronics and Analytics, Analytics (ZEA-3), Forschungszentrum Jülich GmbH, Jülich, 52428, Germany

[5] Institute of Soil Science, Chinese Academy of Sciences, Nanjing, 210008, China

*Corresponding author

Current address: Department of natural resources and environmental sciences, University of Illinois at Urbana-Champaign, Urbana, IL, 61801, USA

Xiaoqian Jiang, Email: jxq1204@illinois.edu, Telephone: (1) 2172812470

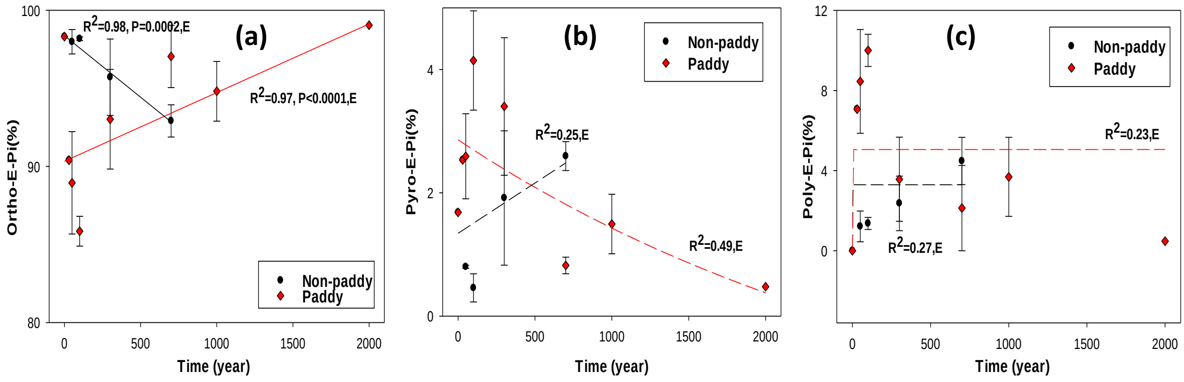

Fig. S1 Impact of land-use duration on the proportion (%) of (a) orthophosphate (Ortho), (b) pyrophosphate (Pyro), (c) polyphosphate (Poly) to total inorganic P.

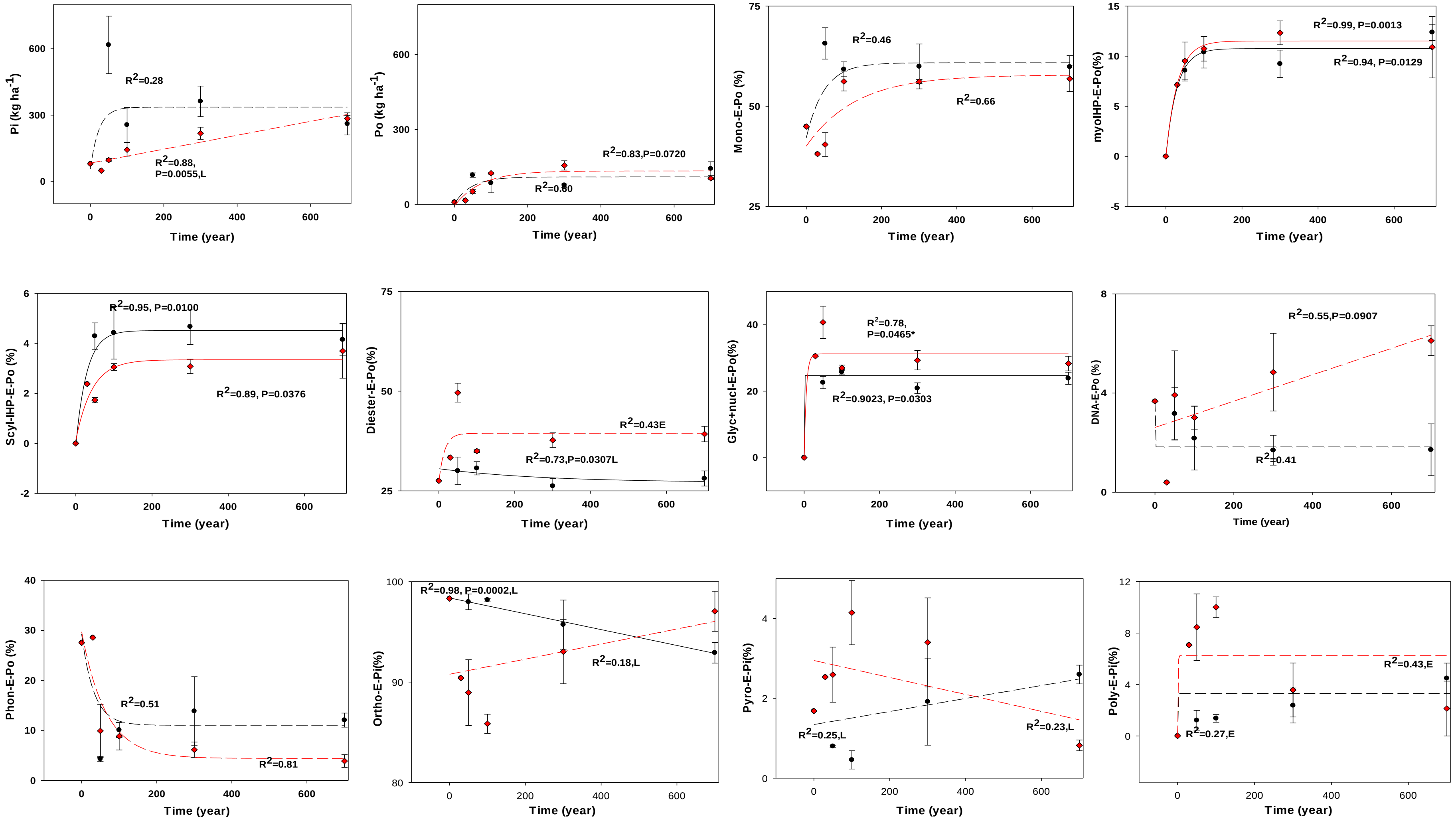

Fig. S2 The comparison of Fig. 1, 2, S2 with only 700-year Paddy (red lines) and non-paddy (black lines) chronosequence.

The determination of the extraction method and delay time for solution ^{31}P-NMR spectroscopy

Phosphorus was extracted by shaking 1g of air-dried soil with 10 mL of a solution containing 0.25 M NaOH and 0.05 M Na_2EDTA for 16h, 4h, and 4h with twice extraction, followed by centrifugation at 10,000×*g* for 30 min. The ^{31}P-NMR results showed that the highest concentration of organic P was extracted with the extraction time of 4h.

The NaOH-Na_2EDTA extractant of 100-year-old paddy topsoil (top layer 0-9 cm) was tested by different repetition delay 0.5 s, 2.6 s, 8.0 s, and 10.5 s for solution ^{31}P-NMR spectroscopy. The sample was in the fridge for 3 weeks before the measurement to make sure all the hydrolysis of the sample is finished. We found a little difference among 2.6 s, 8.0 s, and 10.5 s. Therefore, 2.6 s of repetition delay is long enough for the relaxation for the stable sample.

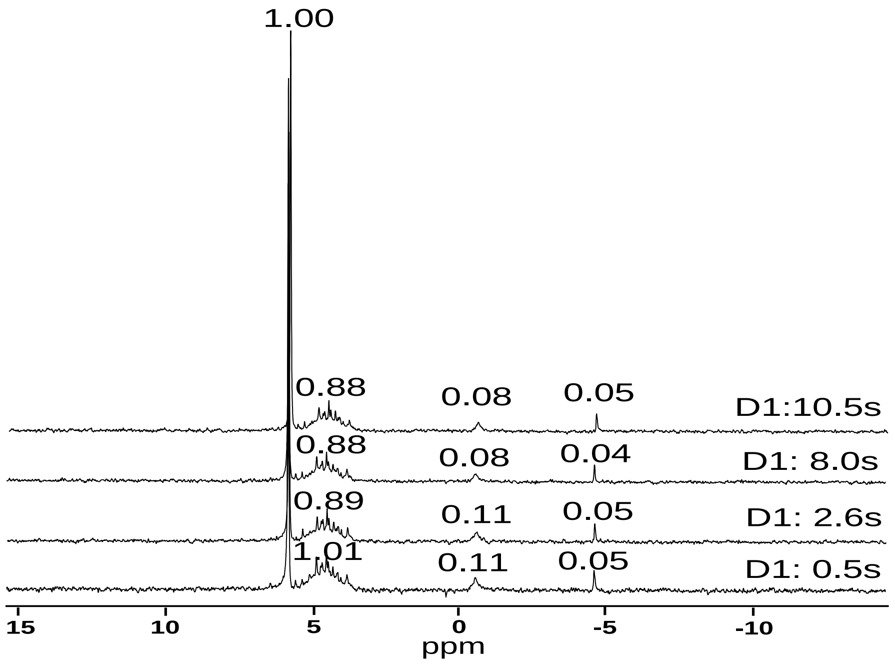

Fig. S3 Solution ^{31}P NMR spectra of NaOH-Na_2EDTA extracts of paddy soil (top layer 0-9 cm) with 100 year rice cultivation with different repetition delay (D1: 0.5, 2.6, 8.0, and 10.5 s, respectively). The area of orthophosphate was standardized to 1.00 and the numbers on the peaks means the area of corresponding peaks.

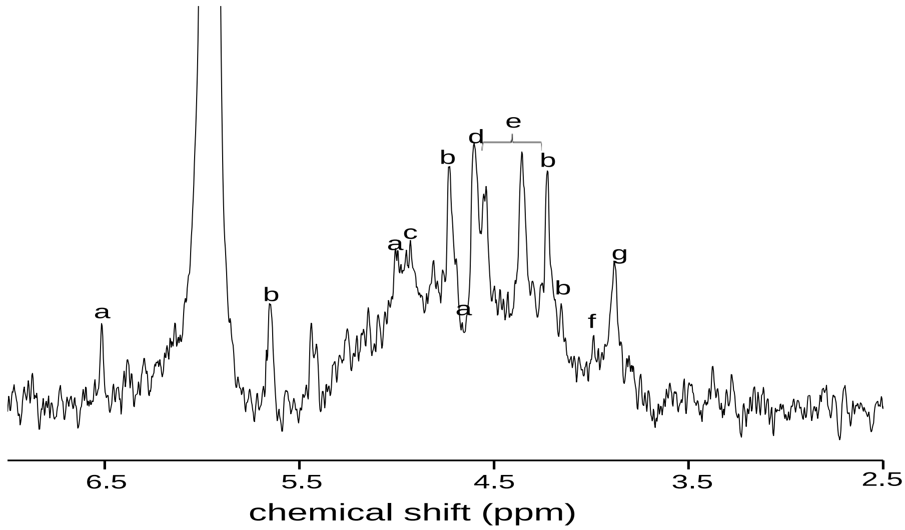

Fig. S4 The solution ^{31}P NMR spectrum of a NaOH-Na_2EDTA extract of the 100-year-old paddy soil. The spectrum shows only the phosphate monoester region and is truncated vertically. The signals were assigned as follows: a, chir*o*-inositol hexakisphosphate 4e/2a (chiro1); b, myo-Inositol hexakisphosphate (myoIHP); c, α glycerophosphate (α-glyc); d, β glycerophosphate (β-glyc); e, mononucleotide; f, choline phosphate (Pchol); g, scyllo-inositol hexakisphosphate (scylloIHP).

Table S1 Kinetic parameters of the mono-exponential model (Eqn 1: $X_t = (X_e - X_0) \times (1 - e^{-kt}) + X_0)$ calculated for paddy organic P and non-paddy inorganic and organic P.

Parameters	Unit	*Xo*	*Xe*	*k*	Accumulation$^{\alpha}$ rate$_{80}$$^{\alpha}$ (1 yr^{-1})	Time to steady$^{\beta}$ (years)
Paddy P_o	kg/ha	--	130.1	0.0143	0.9490	194
NP Pi	kg/ha	57.7	336.2	0.0427	6.3488	85
NP P_o	kg/ha	3.5	110.6	0.0214	1.1536	144
k+SD						
Paddy P_o	kg/ha	--	130.1	0.0212	1.4069	149
NP Pi	kg/ha	57.7	336.2	0.1439	21.3955	34
NP P_o	kg/ha	3.5	110.6	0.0435	2.3450	87
k-SD						
Paddy P_o	kg/ha	--	130.1	0.0074	0.4911	294
NP Pi	kg/ha	57.7	336.2	-0.0585	-8.6979	--
NP P_o	kg/ha	3.5	110.6	-0.0007	-0.0377	--

k, rate constant; X_0, concentration at time point zero; *Xe*, equilibrium concentration; SE, standard error.

[α] Averaged for the cultivation period until 80% of *Xe* were reached.

[β] Defined as annual increase <0.1% of absolute value of the respective parameter.

Table S2 Kinetic parameters of the mono-exponential model (Eqn 1: $X_t = (X_e - X_0) \times (1 - e^{-kt}) + X_0)$ calculated for different phosphorus (P) pools in 700 years paddy management).

Parameters	Unit	k±SE	X_0	X_e	Accumulation rate$_{80}$$^{\alpha}$ (1 yr^{-1})	Time to steady-state$^{\beta}$ (years)	$R^{2§}$
Po	kg/ha	0.0137±0.0085	--	134.4	0.9392	200	0.83
Poly	%	1.1368	0	6.2	3.529	7	0.43
P-mono	%	0.0076±0.0091	40.1	57.8	0.1099	129	0.66
Phon	%	0.0156±0.0099	29.7	4.4	--	--	0.81
Myo-IHP	%	0.0329±0.0048	0	11.5	0.1885	108	0.99
Scyllo-IHP	%	0.0234±0.0105	0.1	3.3	0.038	135	0.89
Gly+nucl	%	--	0	29.5	--	--	0.81

k, rate constant; X_0, concentration at time point zero; Xe, equilibrium concentration; SE, standard error.

$^{\alpha}$ Averaged for the cultivation period until 80% of Xe were reached.

$^{\beta}$ Defined as annual increase <0.1% of absolute value of the respective parameter.

Table S3 Chemical shift of peaks detected in ^{31}P-NMR spectra of NaOH-Na_2EDTA samples.

Category[a]	P Form or Compound Class	Chemical Shift (ppm)
Inorganic P		
	Orthophosphate	6.0
	Pyrophosphate	-4.37±0.14
	Polyphosphate	-6.70±0.40, -10.78±0.49, -12.88±0.57, -15.19±0.93, -18.33±0.44, -23.41±1.77,-32.79±0.82, -28.88±0.87, -7.94±1.05, -42.22±1.43, -44.36±0.28,-47.23±0.80
Organic P	Phosphonates	9.50±0.76, 12.28±0.84, 15.12±0.49, 18.56±1.15, 21.26±0.85, 24.57±1.16, 27.50±0.87, 30.66±1.06, 34.81±0.79, 38.79±0.96, 42.10±0.83, 46.18±0.79, 48.80±0.48
Orthophosphate Monoesters		
	myo-IHP	4.16±0.05, 4.24±0.04, 4.74±0.05, 5.66±0.05
	scyllo-IHP	3.89±0.07
	α-glyc	4.96±0.05
	β-glyc	4.61±0.05
	nucl	4.26±0.03, 4.36±0.03, 4.46±0.03, 4.56±0.03
P-mono-other		
	Chiro-IHP	4.68±0.03, 5.06±0.05, 6.50±0.09
	Pchol	3.97±0.03
	Unknown	3.57±0.13, 3.77±0.04, 4.81±0.07, 5.06±0.07, 5.27±0.05, 5.46±0.03, 6.76±0.08, 6.22±0.05,6.39±0.06, 6.53±0.02
Orthophosphate diesters-- DNA		-1.05±0.16, -0.54±0.11

[a]myo-inositol hexakisphosphate (myo-IHP), scyllo-inositol hexakisphosphate (scyllo-IHP), D-*chiro*-inositol hexakisphosphate 4e/2a (chiro1), α-glycerophosphate (α-glyc), β-glycerophosphate (β-glyc), mononucleotides (nucl), choline phosphate (Pchol), unkown peaks, other P-monoesters (P-mono-other) and deoxyribonucleic acid (DNA).
